# Force-Based Characterization of the Wetting Properties of LDPE Surfaces Treated with CF_4_ and H_2_ Plasmas

**DOI:** 10.3390/polym15092132

**Published:** 2023-04-29

**Authors:** Cihan Aktas, Osman Polat, Mohamadreza Beitollahpoor, Melika Farzam, Noshir S. Pesika, Nurettin Sahiner

**Affiliations:** 1 Department of Chemical, Biomolecular and Materials Engineering, University of South Florida, Tampa, FL 33620, USA; 2 Chemical and Biomolecular Engineering Department, Tulane University, New Orleans, LA 70118, USA; 3 Department of Ophthalmology, Morsani College of Medicine, University of South Florida, 12901 Bruce B. Downs Blvd, MDC21, Tampa, FL 33612, USA; 4 Department of Chemistry, Faculty of Science and Arts & Nanoscience, Technology Research and Application Center (NANORAC), Canakkale Onsekiz Mart University, Terzioglu Campus, 17100 Canakkale, Turkey

**Keywords:** LDPE, plasma modification, surface texture, wetting properties of LDPE, lotus effect

## Abstract

Low-density polyethylene (LDPE) films are widely used in packaging, insulation and many other commodity applications due to their excellent mechanical and chemical properties. However, the water-wetting and water-repellant properties of these films are insufficient for certain applications. In this study, bare LDPE and textured LDPE (T-LDPE) films were subjected to low-pressure plasmas, such as carbon tetrafluoride (CF_4_) and hydrogen (H_2_), to see the effect of plasma treatment on the wetting properties of LDPE films. In addition, the surface of the LDPE film was textured to improve the hydrophobicity through the lotus effect. The LDPE and T-LDPE films had contact angle (θ) values of 98.6° ± 0.6 and 143.6° ± 1.0, respectively. After CF_4_ plasma treatments, the θ values of the surfaces increased for both surfaces, albeit within the standard deviation for the T-LDPE film. On the other hand, the contact angle values after H_2_ plasma treatment decreased for both surfaces. The surface energy measurements supported the changes in the contact angle values: exposure to H_2_ plasma decreased the contact angle, while exposure to CF_4_ plasma increased the contact angle. Kinetic friction force measurements of water drops on LDPE and T-LDPE films showed a decrease in friction after the CF4 plasma treatment, consistent with the contact angle and surface energy measurements. Notably, the kinetic friction force measurements proved to be more sensitive compared to the contact angle measurements in differentiating the wetting properties of the T-LDPE versus 3× CF_4_-plasma-treated LDPE films. Based on Atomic Force Microscopy (AFM) images of the flat LDPE samples, the 3× CF4 plasma treatment did not significantly change the surface morphology or roughness. However, in the case of the T-LDPE samples, Scanning Electron Microscopy (SEM) images showed noticeable morphological changes, which were more significant at sharp edges of the surface structures.

## 1. Introduction

Polymers are crucial materials in various fields, such as packaging, adhesives, automotive and even the biomedical field [1]. Because of their inert nature and desired mechanical properties with tunable functional properties, polymers have replaced traditional engineering materials, such as metals and wood, and have been used in many forms and shapes, such as films, disk, plates, wire, foams, etc. [2,3]. Low-density polyethylene (LDPE) is one of the most widely used polymers in several industries, and its production is well established, easy and cheap [4,5]. Although LDPE has superior thermal, electrical and mechanical properties, it possesses a low surface free energy that affects its wettability and adhesion. LDPE similar to most of the commodity polymers are inert, and there are several contamination layers on the surface, e.g., low-molecular-weight oligomers, additives, release agents, oil, etc. [6,7,8]. Those contaminations make the bonding on the surface of LDPE even more challenging [9]. Most of the industrial and biomedical applications require additional modifications of the polymer surfaces [9,10]. By changing the surface properties, it is possible to improve the wettability, printability and biocompatibility and, hence, enhance the applications of the polymers [11,12].

There are several methods available for the surface modifications of polymers. For example, Shenton et al. used atmospheric pressure plasma to increase the adhesive property of LDPE and polyethylene terephthalate (PET) [9]. Cheng et al. used the vapor phase deposition-initiated roll-to-roll method to modify cellulose chromatography paper [13]. Nejati et al. reported using lasers to functionalize the surface of carbon to attach silver nanoparticles on it [14]. Strobel and his coworkers exposed polypropylene (PP) to flames to improve its wettability [15]. Netravali and his colleagues used pulsed argon ion beams to increase the hydrophilicity of ultra-high-strength polyethylene fibers [16]. Further, even wet chemical techniques are available to functionalize the surface of crystals, such as gallium phosphide crystalline [17].

The low-pressure, cold plasma technique has many advantages for the surface modification of polymers [18,19,20,21]. Plasma treatment alters only a few tens to hundred angstroms of the samples’ surface, while the bulk material remains unchanged [22]. Plasma gas treatment shows better homogeneity on surface modifications of polymers than corona discharge or flame exposure methods [18,23]. The treatments after the plasma process show better uniformity on the treated sample [24,25]. Furthermore, the exposure time can be kept noticeably short [26], and the parameters can be adjusted well and tuned with variable plasma gases and do not require any drying processes, such as wet chemical processes and solvent removal [27]. The plasma gas technique is ecological when the absence of chemical waste is considered [28]. Since it is a dry process, plasma treatment affords significant advantages for industrial applications.

In this study, commercially available LDPE and physically altered LDPE films (i.e., microtextured, as shown in Figure S1) were treated with CF_4_ and H_2_ plasmas. The plasma treatments were performed multiple times, e.g., up to three times, and the changes in the surface energy, water contact angle and roll-of angle were measured. The surface topography of textured LDPE (T-LDPE) samples was examined with the help of scanning electron microscopy (SEM, Hitachi 3400 electron microscope, Santa Clara, CA, USA), and the wetting properties of the surfaces were characterized by contact angle and sliding angle measurements, as well as by force-based friction measurements between a water drop and the films. This study shows the combined effects of the low-pressure plasma treatment and the surface texturing method on LDPE films.

## 2. Materials and Methods

### 2.1. Plasma Treatment

All the experiments for the capacitively coupled plasma gas treatments were conducted via a low-pressure radio frequency (RF,13.56 MHz) plasma generator instrument (Femto AR-PC, Diener Electronic, Ebhausen, Germany). The low-pressure chamber of the plasma generator was evacuated by a rotary vane pump (16 m^3^/h, Trivac D16BCS, Leybold Vakuum GmbH, Köln, Germany), and the pressure was monitored using a Pirani sensor. The inlet gas was introduced into the chamber at a flow rate of 1 mL/min by the mass flow controller of the plasma generator. The optimal working conditions of the plasma generator were set at 0.3 mbar, and the pressure was kept constant at that value during the plasma gas exposure. In the treatments of LDPE, H_2_ and CF_4_ gas plasmas were used. To dispose of the undesired gases from previous runs and the accumulated air from the waiting time, the plasma generator ran empty for 15 min prior to each plasma treatment. To determine the optimum parameters for the employed plasma gases, the LDPE samples were exposed to plasma gases for 2, 4, 6, 8, 10 and 15 min. For each time duration, the plasma power was varied as 30, 90 and 150 W values. After treatments, the water contact angles (CAs) were measured via an optical tensiometer (Biolin Scientific, Attention Theta Flex, Phoenix, AZ, USA). A 150 W, 15 min exposure resulting in the highest CA value for the CF_4_ treatment was chosen, whereas the lowest-CA-value resulting treatment from H_2_ treatments was set with the optimum parameters of 30 W and 8 min.

To see the difference in single and multiple plasma gas treatments, samples were treated one time and three times with the same plasma gas. After treatments, the wetting properties, such as surface free energy, CA, sliding angle and water drop friction, were measured.

### 2.2. Surface Texturing of LDPE

Laser machining (CAJO, Technologies, New Orleans, LA, USA) was used to create a square lattice of circular holes (100 µm in diameter with 150 µm center-to-center spacing) on stainless steel. The latter served as a mold to create the textured LDPE films. In a typical molding step, the stainless-steel mold was preheated on a hotplate to 140 °C. A smooth LDPE film was placed on the mold and allowed to melt. Once the LDPE film turned translucent, a Teflon roller (McMaster-Carr, Douglasville, GA, USA) was used to compress the film so as to ensure that the liquid LDPE entered the holes in the stainless-steel mold. The latter was then cooled to room temperature, and the LDPE film was peeled off the surface, resulting in the textured LDPE film.

### 2.3. Measurements of Wetting Properties Using Contact Angles

The effect of functionalization via plasma treatment was assessed by the change in the wetting properties of the LDPE plate. The characterization of the plasma-treated samples’ CA values was measured via an optical tensiometer. For the CA and surface free energy measurements, the sessile drop method was utilized. A 6 µL volume of liquid dropped on the surface of the sample was used, and 332 frames were recorded in 10 s.

In the sliding angle measurements, a volume of 100 µL distilled (DI) water was used. The sample holder was tilted at a rate of 90° per minute until the droplet slid off the sample surface. During the tilting, the droplets were captured at 5.5 frames per second (FPS), and the changes in CA on both sides of the drop were also recorded to measure the contact angle hysteresis (CAH). The CA, surface free energy and sliding angle CAH results were presented as the average of three different measurements. For the analysis of wetting properties, a software (One Attention from Biolin Scientific, Phoenix, AZ, USA, version 4.2.0) was used.

The OWRK (Owen, Wendt, Rabel and Kaelble) Model was utilized to evaluate the surface free energy of the polymer [29,30,31,32]. This model uses dispersive and polar force components of the material to calculate the surface free energy. The following equation, Equation (1), was used to determine the samples surface tensions.

(1) $${\left(\gamma_{sv}^{d}\gamma_{lv}^{d}\right)}^{\frac{1}{2}}+{\left(\gamma_{sv}^{p}\gamma_{lv}^{p}\right)}^{\frac{1}{2}}=0.5+\gamma_{lv}\left(1+\cos\left(\theta_{Y}\right)\right)$$

where $\gamma_{sv}^{}$ and $\gamma_{lv}^{}$ are the surface tensions of the solids and liquids, respectively; superscripts “*d*” and “*p*” are the dispersive and polar force components; and, finally, *θ_Y_* is the contact angle of the liquid. Previous studies show using water and diiodomethane produces the most accurate results [31,33]. For that reason, DI water and diiodomethane were used for the calculation of dispersive and polar force components.

### 2.4. Measurements of Wetting Properties using Force-Based Friction Measurements

A nanotribometer (UMT Multi-specimen Test system, Bruker (formally CETR), San Jose, CA, USA) with a sensitivity of ± 1 µN and a force range of ±10 mN was used to measure normal and lateral friction forces. The protocol developed by Beitollahpoor et al. [34] was followed. A total of 20 µL water drops were placed on a copper ring probe, with an inside diameter of 2.3 mm, and covered by a PDMS layer on the top so as to prevent each water drop from passing through the probe during compression of the drop on the surfaces (Figure S2). In a typical run, the water drop held by the ring probe was brought into contact with the SH surface at the velocity of 2 mm/s. Once the drop touched the surface, a predetermined preload was applied on the drop for 20 s to allow the solid/liquid interface to equilibrate (resting time) within the static regime. In the next step, the ring probe began to move laterally, thereby sliding the drop on the surface under a constant load, at a velocity of 0.1 mm/s for 30 s. The feedback controller of the instruments ensured that the load was maintained at the desired value throughout this step. In the transition step, while the ring was moving, the water drop remained pinned on the SH surface, and the static friction increased. Typically, the highest friction force was obtained at the very moment the receding edge of the water drop began to slide, i.e., the static friction force, $F_{∥}^{static},$ or the threshold force. The transition regime was followed by the kinetic regime, in which the water drop slid while overcoming the dynamic friction force, $F_{∥}^{kinetic},$ on the SH surface. The drop was finally pulled off of the surface in the last step. Generally, if the surface is homogenous and void of defects, $F_{∥}^{static}$ is greater than $F_{∥}^{kinetic}$. Each run was repeated at least five times, and the error bars correspond to the standard deviations.

## 3. Results

### 3.1. Contact Angle and Surface Free Energy Measurements

The effect of two different plasmas, CF_4_ and H_2_, on the surface of LDPE was studied. For these two different LDPE surfaces, smooth and textured with microstructures were employed. Plasma treatment of the surfaces with either H_2_ or CF_4_ rendered the LDPE surfaces more hydrophilic or hydrophobic with respect to a virgin LDPE surface. The effect of multiple plasma treatments (1× or 3×) was also investigated to determine whether further modification after the first plasma treatments was possible.

**Figure 1:**
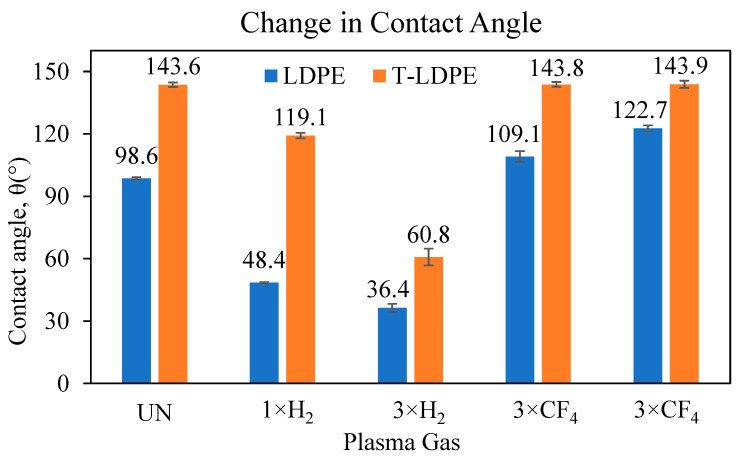
Contact angle (CA) values of LDPE and T-LDPE samples upon multiple H_2_ and CF_4_ plasma treatments.

The CA measurements were determined right after the plasma treatment of LDPE with CF_4_ and H_2_ plasma gases. Figure 1 shows the change in the CA of H_2_- and CF_4_-plasma-gas-treated samples.

After texturing the LDPE films, T-LDPE, the CA values increased drastically from 98.6° ± 0.6 to 143.6° ± 1.0. Since LDPE is inherently hydrophobic, the addition of surface roughness and texture is expected to increase the CA value. In this case, the surface texturing traps air pockets, and water drops remain in the Cassie–Baxter state [35]. After functionalization via CF_4_ and H_2_ plasma treatments, the wetting behavior of the smooth LDPE films showed a higher change than the T-LDPE samples. Upon H_2_ plasma treatment, the generation of radicals and species, such as H^+^, H_2_^+^ and H_3_^+^, the functional groups on the surface of LDPE, reacts with air molecules and gases upon exposure to the atmospheric gases after the plasma treatment [36,37]. This can lead to the formation of more polar groups, such as double carbon bonds, hydroxyl, carboxyl, carbonyl, peroxide, ester and ether on the surface of the LDPE samples, which resulted in lower CA values, e.g., from 98.6° ± 0.6 to 36.4° ± 2.0 after the 3rd H_2_ plasma treatment. This hydrophilicity is attributed to the increased interaction of the newly formed functional groups on the surface of LDPE with water molecules [38,39,40]. The T-LDPE samples followed a similar behavior, and their CA values changed from 143.6° ± 1.0 to 60.80° ± 4.0 after the 3rd H_2_ plasma treatment. A reason for this could be the functionalization of additional surface groups after repeated H_2_ plasma treatments. These results show that the chemical composition of the surfaces also plays a significant role in the surface wetting behavior, in addition to the surface morphology, emphasizing the availability of appropriate functional groups [41,42,43].

It was reported that the surface of the LDPE samples could be fluorinated upon CF_4_ plasma treatment [41]. After introducing the C–CF, CF, CF_2_ and CF_3_ groups by means of CF_4_ plasma treatments, the hydrophobicity of the materials increased [44,45,46]. The main reason for the increased hydrophobicity of fluorinated surfaces is the lower-density packing of fluorocarbons on those surfaces, which causes weaker van der Waals interactions with water [47]. Upon three consecutive CF_4_ plasma treatments, the contact angle values for LDPE samples increased from 98.6° ± 0.6 up to 122.7° ± 1.4. On the other hand, the hydrophobicity of T-LDPE samples did not change significantly, as it was found to change 143.6° ± 1.0 to 143.9° ± 1.6 after the 3rd CF_4_ plasma treatment. These results indicate that the polar force component of T-LDPE samples was almost unchanged after the CF_4_ plasma treatment. The surface area of the T-LDPE in contact with the DI water droplet was much smaller in the Cassie–Baxter state in comparison to the LDPE with smooth surfaces, hence the adhesive forces, and this could be a reason for the unchanged contact angle values. Since the CA measurements were not sufficiently sensitive to detect changes in the wetting properties of the T-LDPE samples before and after CF_4_ plasma treatment, a force-based measurement developed by Beitollahpoor et al. [34] was used and is presented in Section 3.3.

**Figure 2:**
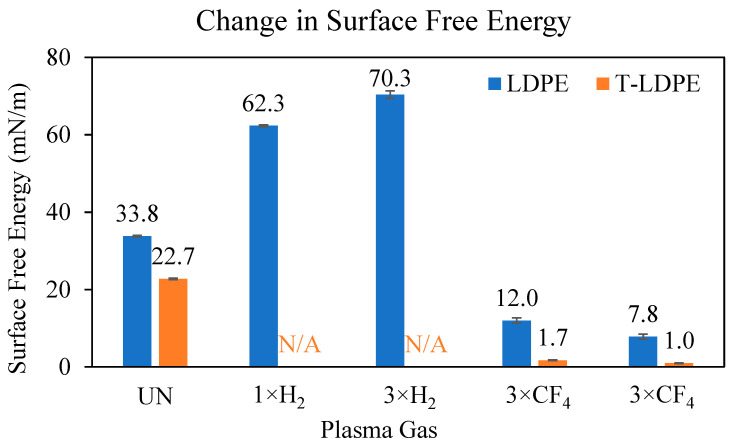
Change in surface free energies of LDPE samples and T-LDPE samples.

The surface free energies of the samples were also measured. Figure 2 depicts the changes of the surface free energies of LDPE samples after plasma treatments.

Since the measurements were determined with DI water and diiodomethane, the surface energy measurements can provide insight not only into the change in the polar force component, but also the change in the dispersive force component of the plasma-treated LDPE and T-LDPE samples. The CA measurements resulted in an increase in hydrophilicity after H_2_ plasma treatments for LDPE and T-LDPE. Therefore, the increase in the surface free energies was expected after H_2_ plasma treatments. The surface free energy of LDPE increased from 33.8 ± 0.2 mN/m to 70.3 ± 1.0 mN/m after the 3rd H_2_ plasma treatment. Unfortunately, the diiodomethane drops formed unmeasurable shapes on the surface of T-LDPE samples after H_2_ plasma treatments; therefore, the surface free energy of these samples could not be measured.

CF_4_ plasma treatments of both the LDPE and T-LDPE samples showed a decrease in surface free energies. These results were expected for LDPE samples, since the fluorination increased the hydrophobicity of the surface of the LDPE samples. The surface free energies of LDPE samples dropped from 33.8 ± 0.2 mN/m to 7.8 ± 0.7 mN/m after being treated 3 times with CF_4_ plasma. Similarly, T-LDPE samples also dropped from 22.7 ± 0.2 mN/m to 1.0 ± 0.1 mN/m after the 3rd CF_4_ plasma treatment. The surface free energy measurements show that the attraction from dispersive force component of both LDPE and T-LDPE surfaces decreased after the CF_4_ plasma treatments.

### 3.2. Sliding Angle Measurements

Any chemical modifications on the sample surface also affects the necessary incline in the tilting angle to slide the droplets off the surface of LDPE and T-LDPE films. The tilting angle of the stage is referred to as the cradle angle, θ_c_. Besides the sliding angles, the contact angle hysteresis (CAH) was also measured. During the tilting of the stage, gravitational force cause deformation in the shape of the droplets. This deformation leads to two results: (1) an increase in CA on one side of the droplets, which is called the advancing contact angle, θ_a_, and (2) a decrease in CA on the other side of the droplets, known as the receding contact angle, θ_r_. The difference between the advancing and the receding contact angle is the CAH [32,48,49]. On hydrophobic surfaces, the sliding occurs at low θ_C_ values, and the deformation in the shape of the droplets is usually very low. When the CA of a surface results in a value greater than 150°, the surface is called super hydrophobic. The CAH values of super-hydrophobic surfaces are close to 0°. The CAH measurements are summarized in Table 1, along with the values of various angles: the sliding angle (α), advancing contact angle (θ_a_) and receding contact angle (θ_r_) for LDPE and T-LDPE films after plasma gas treatments.

H_2_-plasma-treated LDPE and T-LDPE samples show higher CAH values because of increased hydrophilicity. The sliding angle (α) for H_2_-treated LDPE samples increased from 12.5° ± 0.4 to 14.7° ± 1.9. However, H_2_ plasma treatment resulted in a much greater increase for T-LDPE samples. The surface roughness of T-LDPE samples can affect the droplets’ grip on the surface. That effect can increase the droplet resistance against sliding off the T-LDPE surface. For CF_4_-treated samples, on the other hand, the sliding angles of LDPE samples increased from 12.5° ± 0.4 to 27.2° ± 2.7. One reason for that could be the increased nanoscale roughness with the plasma etching, which can lead to drop pinning [41,50,51]. The increased roughness can make the droplets less prone to slide off the surface of LDPE samples. The T-LDPE samples show almost perfect super-hydrophobic behavior after three consecutive CF_4_ plasma treatments. The sliding angle drops from 6.9° ± 0.3 down to 1.7° ± 0.1, and the deformation on droplets is very low when compared to the other samples.

### 3.3. Force-Based Friction Measurements on T-LDPE Surfaces

**Figure 3:**
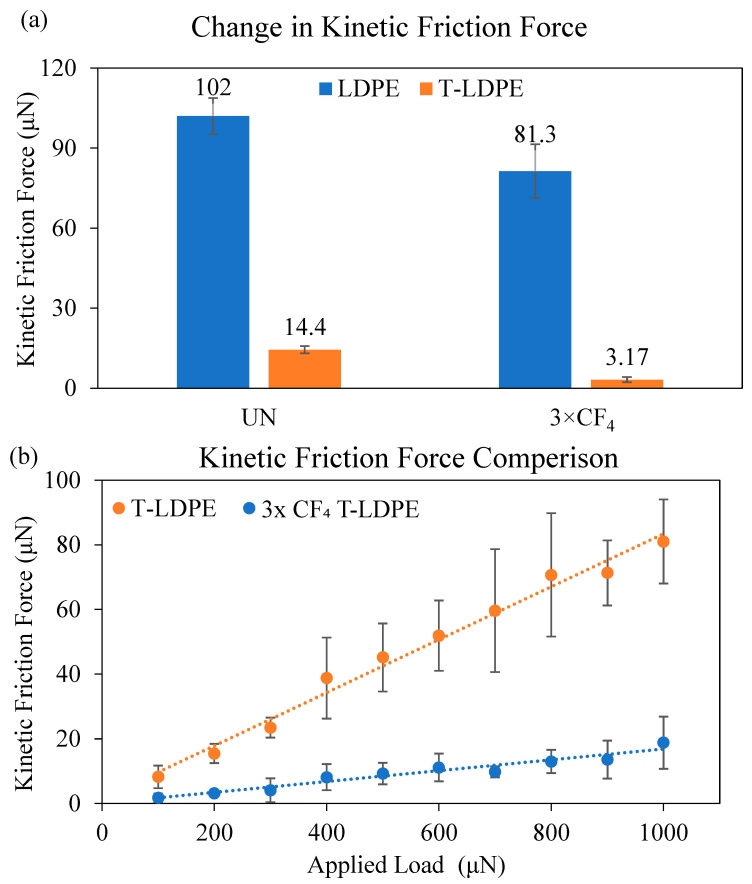
(**a**) Change in the $F_{∥}^{kinetic}$ of LDPE samples and T-LDPE samples after 3× CF_4_ plasma treatment. The applied load on the water drop was maintained at 200 µN. (**b**) Plot of the $F_{∥}^{kinetic}$ versus applied normal load of a 20 µL water drop sheared against an unmodified (red data) and 3× CF_4_-plasma-treated textured LPDE. The shear velocity is 0.1 mm/s. Each experiment was repeated at least 3 times, and error bars represent the standard deviations.

Figure 3a compares the $F_{∥}^{kinetic}$ between a water drop and four surfaces: unmodified smooth LDPE, 3× CF_4_-treated smooth LDPE, unmodified T-LDPE and 3× CF_4_-treated T-LDPE. The force-based measurement could not be performed on the H_2_ plasma-treated samples because the samples were too hydrophilic. As a result of the strong interaction between the water drop and the surface, the ring drop holder was unable to drag the water drop (i.e., the water drop remained pinned on the surface until it detached from the ring drop holder). An applied load equal to the weight of the 20 µL drop (i.e., 200 µN) was maintained during the measurement. The effect of the CF_4_ treatment was apparent on both smooth and T-LDPE surfaces. In the case of the smooth LDPE surface, the $F_{∥}^{kinetic}$ dropped from an average value of 102 µN to 81.3 µN after the CF_4_ treatment. The drop was even more significant on the T-LDPE surface, starting at an average value of 14.4 µN and decreasing to 3.17 µN after the CF_4_ treatment. As demonstrated previously, CA measurements did not show significant differences between the unmodified and CF_4_-plasma-treated T-LDPE samples, even after 3× treatments. Using the nanotribometer, loads ranging from 100 to 1000 µN were applied and maintained on 20 µL drops during shearing against the unmodified and CF_4_-plasma-treated T-LDPE samples. Figure 3b shows a plot of $F_{∥}^{kinetic}$ as a function of the applied preload on a water drop as it is sheared on the T-LDPE surfaces. Under the entire load range of 100–1000 µN, the $F_{∥}^{kinetic}$ on the unmodified T-LDPE (yellow dashed lines) was greater than the $F_{∥}^{kinetic}$ on the CF_4_-plasma-treated T-LDPE (blue dashed lines). The lower $F_{∥}^{kinetic}$ on the CF_4_-plasma-treated T-LDPE is attributed to the lower surface energy provided by the CF_4_ modification (See Figure 2). The water drop remained in the Cassie–Baxter state for both surfaces over the entire applied load range (i.e., the drop did not enter the Wenzel state).

### 3.4. SEM and AFM Images of Altered LDPE Samples

**Figure 4:**
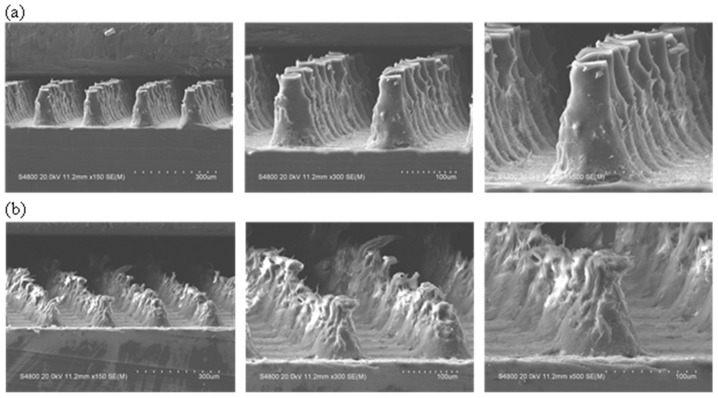
SEM images of (**a**) untreated T-LDPE and (**b**) after three times treatment with CF_4_ (3× CF_4_ T-LDPE) at 3 magnifications.

To see the effect of plasma treatment on surface morphology and roughness, AFM (Bruker Dimension ICON, San Jose, CA, USA) images of the 3× CF_4_-plasma-treated flat LPDE were acquired, and the corresponding images are shown in Figure S3. As can be seen from the AFM images, the surface roughness did not change significantly. However, the SEM images of the T-LDPE samples presented in Figure 4a show significant surface morphological changes, as presented in Figure 4b at the tips of the surface structures, i.e., developing sharp edges after 3× CF_4_ treatments. The plasma process only affects a few tens of nm, maybe up to 50 nm [52,53] of the surface layer of the polymers, depending on the nature of the polymers, such as the crystallinity, glass transition temperature, MW and the extent and the nature of the functional groups. Upon comparison of the SEM images of the T-LDPE samples before and after 3× CF_4_, there is some morphological change in the surface of the T-LDPE samples that occurred due to the 3× CF_4_ treatments.

The low-pressure plasma process plays a very limited role in the physical alteration of the T-LDPE, depending on the plasma parameters, such as exposure time, power, etc.; however, it is possible to induce some slight alteration in the surface morphology of T-LDPE with plasma exposure, while maintaining the main features. As plasma parameters, e.g., power, exposure time, etc., can be controlled, it is possible to find appropriate parameters that do not induce recognizable changes on the surface features of T-LDPE. However, it may be necessary to make chemical modifications on the surface of LDPE, e.g., generation of C-F groups so that the chemical modification is achieved, and this can somehow change the surface morphology. Moreover, etching could play an important role in the surface modification of T-LDPE samples [54,55,56].

## 4. Conclusions

LDPE and T-LDPE samples were treated with successive H_2_ and CF_4_ plasmas. H_2_ plasma treatments caused a decrease in water CA measurements, while CF_4_ plasma treatments resulted in an increase in water CA measurements. After a 3rd H_2_ plasma treatment, the surface free energy value of LDPE increased to 70.3 ± 1.0 mN/m from 33.8 ± 0.2 mN/m, and after a 3rd CF_4_ treatment, the surface free energy value decreased to 7.8 ± 0.7 mN/m. The sliding angle α value of LDPE samples increased from 12.5° ± 0.4 to 14.7° ± 1.9 after the 3rd H_2_ plasma treatment and to 27.2° ± 2.7 after the 3rd CF_4_ treatment. The CA of T-LDPE decreased from 143.6° ± 1.0 to 60.8° ± 4.0 after the 3rd H_2_ plasma treatment and increased to 143.9° ± 1.6 after the 3rd CF_4_ plasma treatment. The surface free energy of T-LDPE decreased from 22.74 ± 0.2 mN/m to 1.0 ± 0.1 mN/m after the 3rd consecutive CF_4_ plasma treatment. The sliding angle of T-LDPE samples increased to 38.2° ± 2.5 from 6.9° ± 0.3 after the 3rd H_2_ plasma treatment and decreased to 1.7° ± 0.1 after the 3rd CF_4_ plasma treatment. The CAH values of the samples increased based on the increases of the hydrophilicity of the surfaces, regardless of the treatment. However, samples with surface features, e.g., T-LDPE, had a lower CAS than their non-textured surfaces, e.g., LDPE with smooth surfaces. After the 3rd consecutive CF_4_ plasma treatment, the friction force between water drops, and LDPE and T-LDPE films dropped from 102 µN to 81.3 µN and from 14.4 µN to 3.17 µN, respectively. These results suggest that the combinatory effect of surface texturing and plasma gas treatments of LDPE provide significant changes in the wetting properties of surfaces, which are potentially advantageous in many applications using this material. The area of application of textured and plasma-treated samples not only include tubing, piping, packing and food and beverage, but also cover biomedical applications.

## Figures and Tables

**Table 1:** Change in sliding angle (α), advancing contact angle (θ_a_), receding contact angle (θ_r_) and contact angle hysteresis (CAH) after plasma treatment of LDPE and T-LDPE samples.

Sample	Sliding Angle, α (°)	Advancing Contact Angle, θ_a_ (°)	Receding Contact Angle, θ_r_ (°)	Contact Angle Hysteresis, CAH = θ_a_ − θ_r_ (°)
LDPE	12.5 ± 0.4	102.0 ± 0.8	80.1 ± 2.7	21.9
T-LDPE	6.9 ± 0.3	130.2 ± 3.5	110.5 ± 4.8	19.7
LDPE, 3× H₂	14.7 ± 1.9	47.6 ± 0.6	7.6 ± 0.5	40
T-LDPE, 3× H₂	38.2 ± 2.5	107.1 ± 3.5	23.2 ± 3.8	83.9
LDPE, 3× CF_4_	27.2 ± 2.7	141.1 ± 1.0	81.1 ± 2.1	59.9
T-LDPE, 3× CF_4_	1.7 ± 0.1	121.2 ± 0.7	118.3 ± 0.9	2.8

## Data Availability

The data presented in this study are available on request from the corresponding author.

## Supplementary Materials

The following supporting information can be downloaded at: https://www.mdpi.com/article/10.3390/polym15092132/s1, Figure S1: LDPE (top left) and T-LDPE (top right) samples. DI water forms different shapes on LDPE (bottom left) and on T-LDPE (bottom right); Figure S2: Optical image of the experimental setup used to measure the kinetic friction between a water drop and a textured LDPE sample. The water drop is 20 µL. The copper ring drop holder is connected to a dual-axis force sensor which allows for simultaneous normal and lateral force measurements.; Figure S3: AFM images using tapping mode of flat LDPE samples (a) before, (b) after 3× CF4 plasma treatment. The surface roughness (Rq) changes from 9.22 ± 3.73 nm to 10.28 ± 3.73 nm. The surface roughness values were obtained by averaging the roughness of 3 random locations on each sample.
