# Supplementary material for: Force-Based Characterization of the Wetting Properties of LDPE Surfaces Treated with CF4 and H2 Plasmas
Source: Polymers (Basel). 2023 Apr 29;15(9):2132. doi: 10.3390/polym15092132 (PMC10181085; doi:10.3390/polym15092132)
Supplement: Supplementary file 1 [file polymers-15-02132-s001.zip › polymers-2351784-supplementary.pdf]

Supporting Information

Force-Based Characterization of the Wetting Properties of LDPE Surfaces Treated with CF_4 and H_2 Plasmas

Cihan Aktas [1], Osman Polat [1], Mohamadreza Beitollahpoor [2], Melika Farzam [2], Noshir S. Pesika [2] and Nurettin Sahiner [1,3,4,*]

[1] Department of Chemical, Biomolecular and Materials Engineering, University of South Florida, Tampa, FL 33620, USA
[2] Chemical and Biomolecular Engineering Department, Tulane University, New Orleans, LA 70118, USA
[3] Department of Ophthalmology, Morsani College of Medicine, University of South Florida, 12901 Bruce B. Downs Blvd, MDC21, Tampa, FL 33612, USA
[4] Department of Chemistry, Faculty of Science and Arts & Nanoscience, Technology Research and Application Center (NANORAC), Canakkale Onsekiz Mart University, Terzioglu Campus, 17100 Canakkale, Turkey
* Correspondence: sahiner71@gmail.com or nsahiner@usf.edu

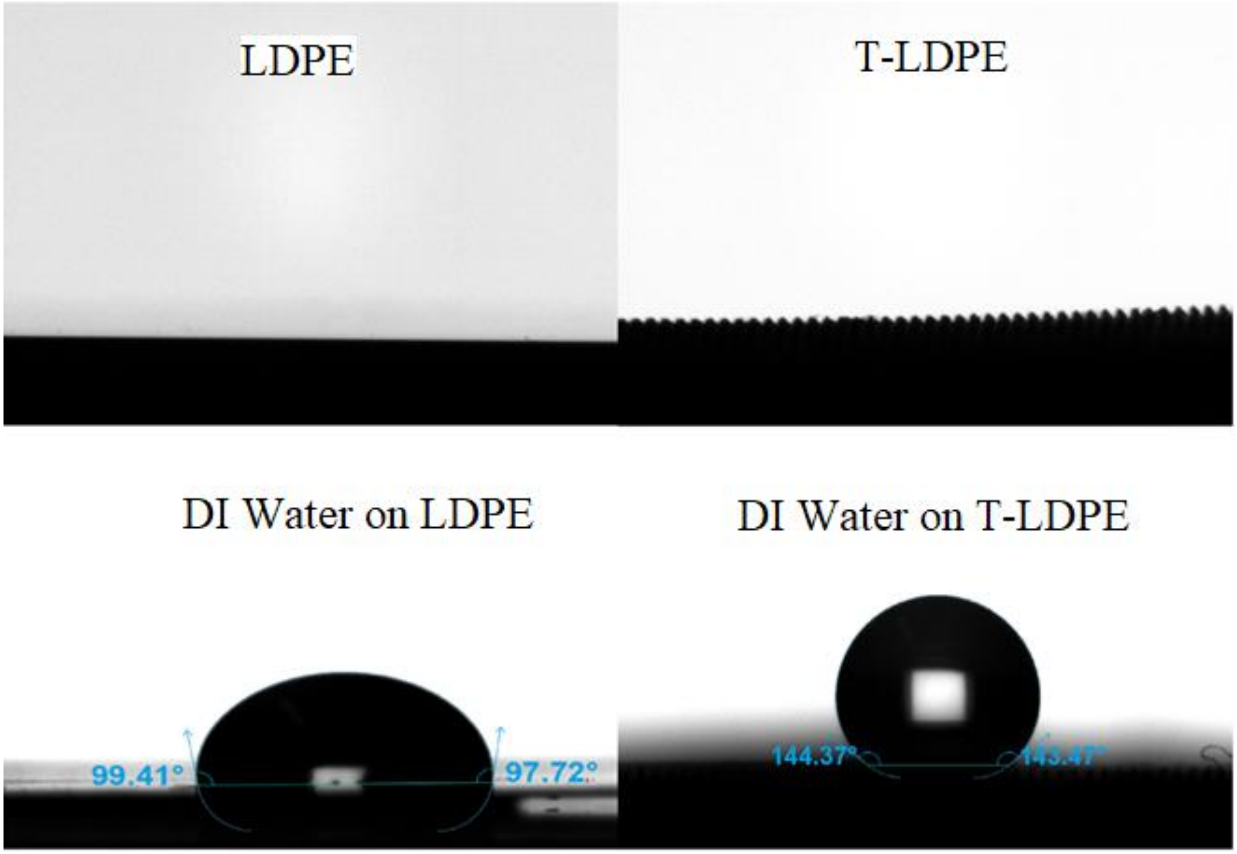

Figure S1. LDPE (top left) and T-LDPE (top right) samples. DI water forms different shapes on LDPE (bottom left) and on T-LDPE (bottom right).

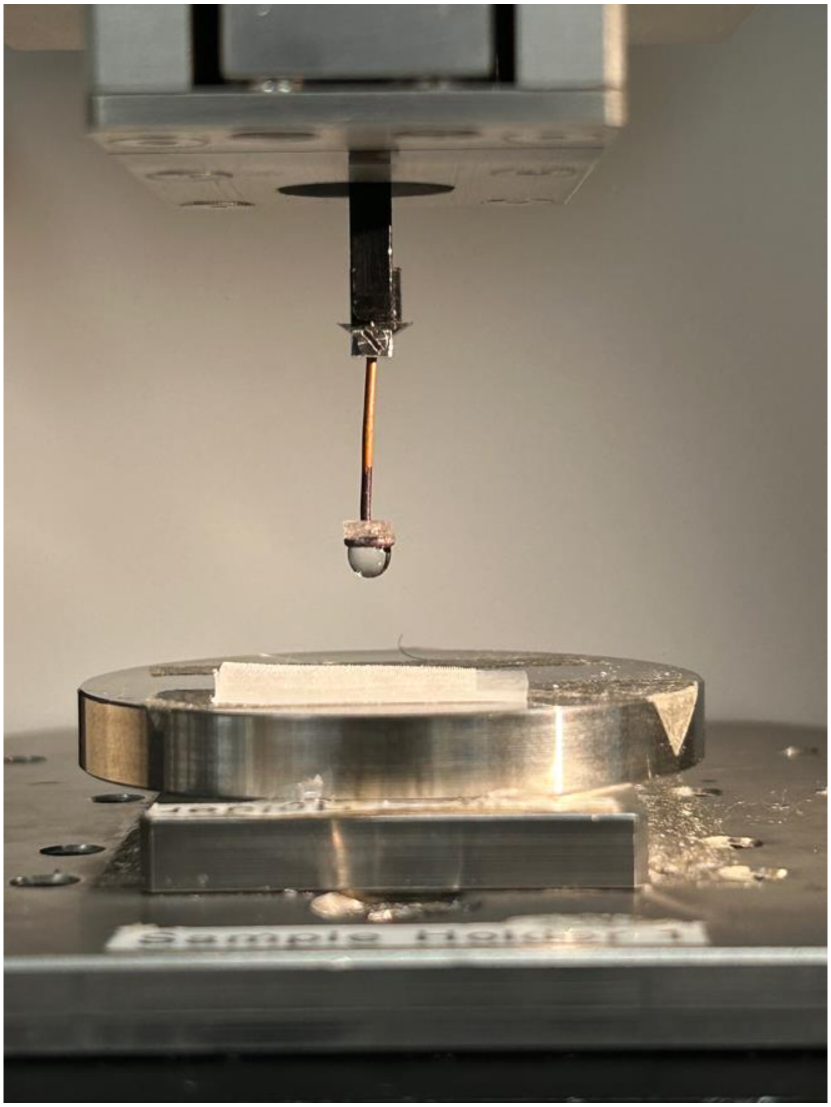

Figure S2. Optical image of the experimental setup used to measure the kinetic friction between a water drop and a textured LDPE sample. The water drop is 20 µl. The copper ring drop holder is connected to a dual-axis force sensor which allows for simultaneous normal and lateral force measurements.

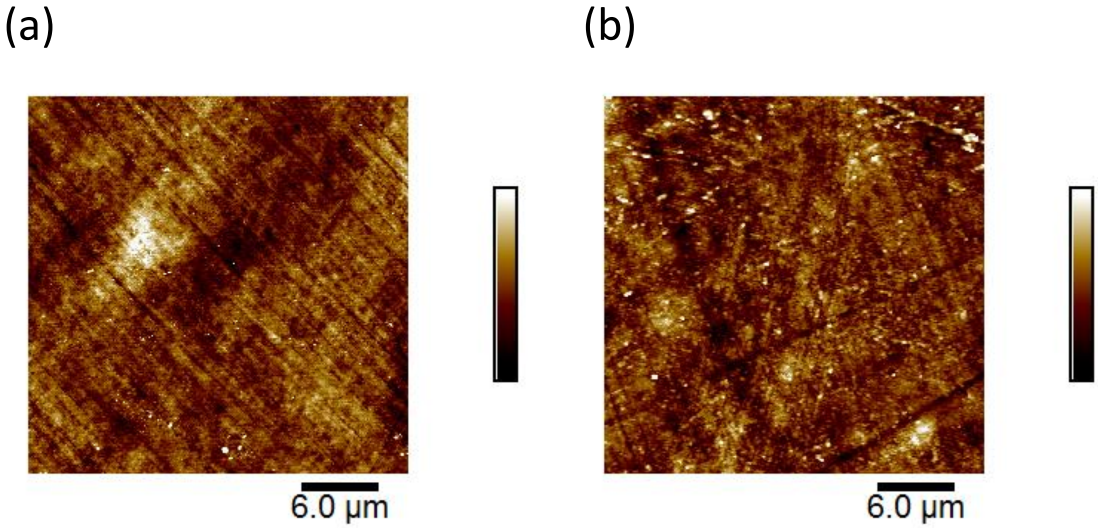

Figure S3. AFM images using tapping mode of flat LDPE samples (a) before, (b) after 3x CF_4 plasma treatment. The surface roughness (R_q) changes from 9.22±3.73 nm to 10.28±3.73 nm. The surface roughness values were obtained by averaging the roughness of 3 random locations on each sample.
